# The Effect of Dietary Intervention on Autosomal-Dominant Polycystic Kidney Disease (ADPKD) Patients on Tolvaptan and Their Quality of Life

**DOI:** 10.7759/cureus.25045

**Published:** 2022-05-16

**Authors:** Ola Tarabzuni

**Affiliations:** 1 Department of Nephrology, McMaster University, Hamilton, CAN

**Keywords:** tolvaptan, quality of life, adpkd, autosomal-dominant polycystic kidney disease, intervention, dietary

## Abstract

Background and objective

Autosomal-dominant polycystic kidney disease (ADPKD) is the most common inherited renal disorder; it affects people of all ethnic groups and is found in up to 10% of patients with end-stage renal disease (ESRD). Dietary intervention is important in people with renal disease, and it has been linked to greater estimated glomerular filtration rate (eGFR) preservation. Tolvaptan, an orally-active nonpeptide, selective arginine vasopressin (AVP) V2R antagonist, was recently licensed in numerous countries for the treatment of ADPKD. The aim of this study was to assess the role of dietary intervention in decreasing the osmotic load on the urine volume and its impact on the quality of life (QOL) of patients with ADPKD on tolvaptan.

Methods

This prospective cohort study was carried out at a Hamilton nephrology genetics clinic. ADPKD patients on well-tolerated doses of tolvaptan for three months were included in the study. Gitelman and Bartter Symptom Health-related QOL questionnaire was used among the study participants.

Results

Our study consisted of nine adult patients with ADPKD who were on a stable dose of tolvaptan therapy. Patients had laboratory values for urine volume, sodium (Na), and urea. No significant difference was found between pre- and post-diet intervention values in 24-hour urine volume (5.9 vs. 5.49 L/d; p=0.423), urine Na (p=0.174), and 24-hour urine urea (p=0.404).

Conclusion

Dietary intervention in ADPKD patients on tolvaptan therapy can play a vital role in improving their QOL. Further research including interventional studies and clinical trials with larger sample sizes is needed to gain deeper insight into the subject.

## Introduction

The most common inherited nephropathy, autosomal-dominant polycystic kidney disease (ADPKD), affects people of all ethnic groups and is found in almost 10% of patients with end-stage renal disease (ESRD). The symptoms of the condition include chronic flank pain, abdominal fullness, and, in severe cases, early satiety, which is characterized by gradual enlargement of the kidneys due to cyst formation. Circulatory hypertension and urologic consequences such as cyst bleeding, extensive haematuria, recurrent urinary tract infections, and nephrolithiasis are all linked to kidney cysts. Extrarenal symptoms of ADPKD include cerebral aneurysms, which can cause catastrophic bleeding if ruptured, liver cysts, colonic diverticular disease, abdominal hernias, and cardiac valve anomalies. ADPKD imposes a tremendous burden on affected patients due to its progressive nature, related concomitant illnesses, and genetic origin [1,2]. ADPKD is the fourth most common cause of ESRD after diabetes, hypertension, and glomerular disease. Its prevalence is estimated to be between 2.7 and 9.3 per 10,000 people [3,4].

Patients with noncommunicable diseases benefit greatly from dietary changes. Dietary intervention is crucial in people with renal diseases, and it has been linked to greater estimated glomerular filtration rate (eGFR) preservation. As a result, many guidelines urge patients to consult a nutritionist early on in the course of the kidney illness to optimize clinical results. A diet low in calories, protein, salt, and phosphate may worsen renal disease-related clinical and metabolic abnormalities and reduce the effectiveness of drug therapy in individuals with advanced kidney disease. Thus, reductions in protein, phosphorus, potassium, and sodium consumption, as well as limiting the fixed acid load, should be evaluated, and adjusted based on patient characteristics, along with strict follow-ups to avoid malnutrition associated with these limits. Another factor to examine is the cause of kidney illness, such as poor glycometabolic management [5-8].

The illness phenotype can be widely diverse even within the same family, resulting in different rates of cyst growth. Dietary factors may contribute to some of the diversity in this rate of renal enlargement, a process that appears to be accelerated by excessive salt and animal-sourced protein. Furthermore, higher urine acid excretion has been reported to speed up cyst growth in experimental models, such as animal studies, and it causes a faster deterioration in kidney function in people with moderately advanced chronic progressive renal disorders, such as ADPKD. High amounts of animal-sourced dietary protein cause acid excretion, although base-producing fruits and vegetables can help to lower it. Increasing fluid consumption can lower plasma levels of arginine vasopressin (AVP), thereby reducing kidney weight (percentage of total body weight) by 27-30%, a result attained by reaching a urine osmolality of 290 mosm/kg H_2_O [9-13].

Tolvaptan, an orally active nonpeptide selective AVP V2R antagonist, was recently licensed in numerous countries for the treatment of ADPKD. Tolvaptan was found to slow down the increase in total kidney volume (TKV) and renal function decrease in two randomized, double-blind, controlled phase 3 trials: TEMPO 3:4 and REPRISE [14]. However, a high frequency of aquaresis-related side effects (thirst, polydipsia, polyuria, and nocturia) was observed in these clinical trials. Despite the fact that contemporary treatment guidelines recommend regular assessments of the quality of life (QOL) in patients receiving tolvaptan, the drug's impact on the patients' QOL has not been investigated systematically and remains unclear [15,16]. The aim of this study was to assess the role of dietary intervention in decreasing the osmotic load on the urine volume and its impact on the QOL of patients with ADPKD on tolvaptan.

## Materials and methods

This prospective cohort study was carried out at an outpatient nephrology genetics clinic. Patients diagnosed with ADPKD on a well-tolerated dose of tolvaptan (45/15 mg, 60/30 mg, or 90/30 mg) for three months were included in the study while patients whose serum Na clearance was <135 mmol/L or >145 mmol/L, and those who were scheduled for dialysis and transplant were excluded from the study. Although patient recruitment was successful, our study was interrupted by the coronavirus disease 2019 (COVID-19) pandemic, and the ability of the patients to attend the clinic to meet with our study dietitian was curtailed. This, unfortunately, led to some patients dropping out (at a rate of 56%). Informed consent was obtained from each participant prior to the initiation of the study. The study has been conducted in alignment with the known ethical research and surveillance recommendations.

After informed consent was obtained, patients were requested to provide a 24-hour urine sample. Urine volume, urea, and Na were measured and laboratory values were recorded. Patients’ history and other physical parameters were obtained from the hospital records and clinic visit notes. Participants met with our clinic dietitian for a dietary intervention. The goal was to reduce the osmotic load by adjusting the amount of water, protein, and salt intake to the level that decreases daily urine osmoles output, which would decrease the volume of urine. The dietitian reviewed a detailed diet history for the patients and provided tailored dietary advice to enable participants to achieve and maintain a moderate protein intake (0.75-1.0 g/kg/day) and to limit sodium intake to 80-100 mmol/day for a duration of three months. A health-related QOL questionnaire was provided to study participants. The questionnaire further consisted of two separate questionnaires: one questionnaire was used among patients with central diabetes insipidus as assessed by the Nagasaki Diabetes Insipidus Questionnaire, and the second one was used in the evaluation of symptoms and health-related QOL in patients with Gitelman/Bartter syndrome.

The data were prepared and entered into Microsoft Excel. Variables were then imported into SPSS Statistics version 26 (IBM, Armonk, NY) for statistical analysis. The Kolmogorov-Smirnov test was applied to analyze the nature of data distribution. Inferential statistics (paired t-test and Wilcoxon signed-rank test) were used to assess the difference between pre- and post-diet intervention lab reports and QOL assessment total scores. The Pearson correlation coefficient was used to evaluate the association between lab readings and QOL. Qualitative variables were expressed as percentages, while quantitative variables were expressed as mean and standard deviation (SD). A p-value <0.05 was considered statistically significant.

## Results

This study looked at the effects of dietary intervention on urine volume, urine Na, urine urea, and QOL. The study population consisted of nine adult patients with ADPKD who were on a stable dose of tolvaptan therapy. Patients had laboratory values for urine volume, Na, and urea. According to our data, there were no significant differences between pre- and post-diet intervention laboratory test results. No significant difference was found in 24-hour urine volume (5.9 vs. 5.49 L/d; p=0.423), urine Na (p=0.174), and 24-hour urine urea, (p=0.404). However, Na showed a 30.21% reduction from pre-diet to post-diet, and the urine score showed a 20% reduction (Table 1).

**Table 1:** Laboratory tests Na+: sodium; SD: standard deviation

	Pre-diet, mean ± SD	Post-diet, mean ± SD	P-value	Percentage change, mean (SD)
Volume (L/d)	5.9 ± 1.59	5.49 ± 2.05	0.423	6.949 (28.93)
Na+ (mmol/L)	201.20 ± 67.12	140.40 ± 119.56	0.174	30.21 (78.12)
Urea (mmol/day)	386.57 ± 116.34	340.14 ± 122.60	0.404	12.01 (5.38)
Urine score	19.63 ± 4.34	20.13 ± 4.45	0.699	20.13 (2.53)

In terms of QOL, no statistically significant changes were found. However, there were reductions in the average scores of general symptoms (30.93%), as well as musculoskeletal (21.92%), renal (6.85%), and gastrointestinal (14.80%) problems. The impact of these symptoms on the QOL was also reduced, but the changes were not found to be statistically significant. The impact of general symptoms showed a 40.42% reduction, followed by the impact on musculoskeletal (35.86%) and renal (8.89%) problems. No reduction was observed in the gastrointestinal symptoms (Table 2).

**Table 2:** Quality of life measures before and after dietary intervention SD: standard deviation

	Pre-diet, mean ± SD	Post-diet, mean ± SD	P-value	Percentage change in average score
General	3.62 ± 1.76	2.50 ± 2.00	0.071	30.93%
Impact	1.88 ± 1.45	1.12 ± 1.72	0.119	40.42%
Musculoskeletal	6.25 ± 4.30	4.88 ± 4.76	0.407	21.92
Impact	4.88 ± 4.22	3.13 ± 3.31	0.259	35.86%
Renal	18.25 ± 3.61	19.50 ± 4.96	0.610	6.85%
Impact	5.62 ± 3.42	5.12 ± 3.04	0.498	8.89%
Gastrointestinal	2.50 ± 2.07	2.13 ± 2.03	0.598	14.80%
Impact	1.63 ± 1.51	1.63 ± 1.60	1.00	0%

The relationship between laboratory findings and QOL scores was analyzed using the Pearson correlation coefficient. There was a significant negative correlation between urine volume and QOL scores of general symptoms (r=-0.845, p=0.008) and musculoskeletal symptoms (r=-0.811, p=0.015), and an inverse correlation was found between the impact of general symptoms and musculoskeletal symptoms on urine volume as well. The relationship between urea in urine and the impact on renal (r=-0.735; p=0.038) and musculoskeletal (r=-0.804; p=0.016) symptoms showed a significant negative correlation (Table 3).

**Table 3:** Correlation between lab findings and quality of life measuring scores (post-diet) Figures in brackets represent p-values Na+: sodium; MSK: muskuloskelatal; GI: gastrointestinal

	General	General impact	MSK	MSK impact	Renal	Renal impact	GI	GI impact
Volume	-0.845 (0.008)	-0.742 (0.035)	-0.811 (0.015)	-0.791 (0.019)	0.554 (0.154)	-0.052 (0.903)	-0.636 (0.090)	-0.270 (0.517)
Na+	-0.772 (0.072)	-0.794 (0.059)	-0.659 (0.155)	-0.822 (0.045)	0.528 (0.282)	-0.078 (0.883)	-0.540 (0.269)	-0.231 (0.659)
Urea	-0.617 (0.103)	-0.506 (0.200)	-0.672 (0.068)	-0.804 (0.016)	0.572 (0.138)	-0.735 (0.038)	-0.227 (0.589)	-0.087 (838)

## Discussion

Our findings revealed that there were no significant differences between pre- and post-diet intervention laboratory test results. No significant difference was found in 24-hour urine volume, urine Na, and 24-hour urine urea. We had expected the decrease in urine volume to be clinically significant, but our findings showed otherwise. This may be due to the small sample size, timing of the administration of the questionnaires, and diet modification status of the participants. Also, because of COVID-19, there was a delay in the period from dietary intervention to the second 24-hour urine collection. We originally planned to have two urine collections post-intervention, but this was not completed due to the limitations imposed by the pandemic. However, Na+ showed a 30.21% reduction from pre- to post-intervention. Reduction in serum Na is a positive aspect of our study results. According to the Canadian Expert Consensus on Assessing Risk of Disease Progression and Pharmacological Management of ADPKD, the salt consumption in these patients should be similar to that of hypertensive patients, which amounts to roughly 5 g of salt or 87 mmol of Na per day. A sodium-restricted diet of 2.4 g/day (100 mmol/day) is recommended for patients treated with tolvaptan, according to the guidelines [17,18]. The need for a sodium-restricted diet for blood pressure regulation in ADPKD was further addressed at the 2015 KDIGO Controversies Conference on ADPKD. Limiting salt consumption could also be beneficial in terms of osmole load [19]. A study, HALT-PKD randomized clinical trials (RCTs) of renin-angiotensin system (RAS) inhibition and blood pressure control conducted in 2017, also reported that Na intake dropped to 3.5-3.8 g/day (145-160 mmol/day) during the trial period [20].

Our study results depicted that in terms of QOL, there was a reduction in the average scores of general symptoms (30.93%), as well as musculoskeletal (21.92%), renal (6.85%), and gastrointestinal (14.80%) problems. This is similar to the results of a Swiss study conducted from 2015 to 2019, which revealed that patients who received tolvaptan treatment reported improved scores in bodily pain and physical functioning after a year of follow-up compared to the general population. At the one-year follow-up, patients without tolvaptan medication had lower scores in general health as compared to the general population, but their scores regarding body pain were higher as compared to the control group. The study illustrates that by using the general SF-36 section of the KDQOL-SF questionnaire, a prospective QOL assessment can be performed in patients continuing with tolvaptan treatment. After the first three months of treatment, we concluded that the medication is well-tolerated with no significant detriment on overall physical or mental health scores. At the follow-up, a patient-reported feedback evaluation of renal disease-specific items demonstrated an increase in patient satisfaction. However, the causes of the higher satisfaction in tolvaptan-treated patients are unknown [2].

The diet prescription used in this trial was designed to mitigate the effect of tolvaptan on the urine volume, and we wanted to assess its impact on the QOL of patients with ADPKD. Taylor et al. state that although none of the individual components of the diet are unique, the restriction of salt and animal-sourced protein intake, as well as increased intake of fruits, vegetables, and water, has never been studied in patients with ADPKD before [21]. Single-component dietary regimes, such as those involving Na, have a relatively low rate of compliance in the general population, and hence it would seem reasonable to assume that adding other elements to the prescription would further impair meaningful adoption. ADPKD patients differ from the general population in this regard. Many people have seen a parent or sibling go through kidney failure, dialysis, and renal transplantation, and they appear to be ready to adhere to medication regimens that may help delay the progression of the disease. The findings of their study revealed that based on the responses to the questionnaire, with the exception of one person, the participants mastered the complexity of meal preparation and record-keeping with relatively minor difficulties [21].

Preclinical research suggests that more precisely defined dietary regimes, such as calorie restriction, time-restricted eating, and ketogenic diets, may decrease disease progression, and the findings of ongoing human clinical studies are keenly anticipated [22]. These dietary interventions have a direct impact on nutrient signaling and substrate availability in the cystic kidney, apart from providing metabolic advantages throughout the body [22]. Tolvaptan, a selective vasopressin V2 receptor antagonist, reduces pain in ADPKD patients with relatively preserved renal function by delaying the increase in kidney volume (a surrogate marker for disease progression), slowing the decline in renal function, and delaying the increase in kidney volume (a surrogate marker for disease progression). Tolvaptan's most prevalent side effects are related to its aquaretic function, and rare occurrences of idiosyncratic hepatitis have been reported. More research is being done to see if the benefits can be sustained over time, if they can be seen in patients with advanced kidney disease, and if they can be translated into improved QOL and cost-effectiveness measures [23]. The restrictions imposed by the COVID-19 pandemic and small sample size were the limitations of our study.

## Conclusions

Based on our findings, diet modification did not bring any additional benefits in terms of QOL or led to any change in urine volume among the study participants. However, dietary intervention in ADPKD patients on tolerated tolvaptan therapy can play a vital role in improving their QOL. Further research including interventional studies and clinical trials on larger sample sizes is needed, which will not only help highlight the importance of dietary modification in ADPKD patients on tolvaptan therapy but also aid in improving their QOL.
